# Diffusion-weighted MRI for predicting pathologic response to neoadjuvant chemotherapy in breast cancer: evaluation with mono-, bi-, and stretched-exponential models

**DOI:** 10.1186/s12967-021-02886-3

**Published:** 2021-06-02

**Authors:** Shiteng Suo, Yan Yin, Xiaochuan Geng, Dandan Zhang, Jia Hua, Fang Cheng, Jie Chen, Zhiguo Zhuang, Mengqiu Cao, Jianrong Xu

**Affiliations:** 1 Department of Radiology, Renji Hospital, School of Medicine, Shanghai Jiao Tong University, No. 160, Pujian Rd, Shanghai, 200127 China; 2 Biomedical Instrument Institute, School of Biomedical Engineering, Shanghai Jiao Tong University, Shanghai, China

**Keywords:** Breast cancer, Diffusion-weighted MRI, Pathologic complete response, Neoadjuvant chemotherapy, Predictive model

## Abstract

**Background:**

To investigate the performance of diffusion-weighted (DW) MRI with mono-, bi- and stretched-exponential models in predicting pathologic complete response (pCR) to neoadjuvant chemotherapy (NACT) for breast cancer, and further outline a predictive model of pCR combining DW MRI parameters, contrast-enhanced (CE) MRI findings, and/or clinical-pathologic variables.

**Methods:**

In this retrospective study, 144 women who underwent NACT and subsequently received surgery for invasive breast cancer were included. Breast MRI including multi-*b*-value DW imaging was performed before (pre-treatment), after two cycles (mid-treatment), and after all four cycles (post-treatment) of NACT. Quantitative DW imaging parameters were computed according to the mono-exponential (apparent diffusion coefficient [ADC]), bi-exponential (pseudodiffusion coefficient and perfusion fraction), and stretched-exponential (distributed diffusion coefficient and intravoxel heterogeneity index) models. Tumor size and relative enhancement ratio of the tumor were measured on contrast-enhanced MRI at each time point. Pre-treatment parameters and changes in parameters at mid- and post-treatment relative to baseline were compared between pCR and non-pCR groups. Receiver operating characteristic analysis and multivariate regression analysis were performed.

**Results:**

Of the 144 patients, 54 (37.5%) achieved pCR after NACT. Overall, among all DW and CE MRI measures, flow-insensitive ADC change (ΔADC_200,1000_) at mid-treatment showed the highest diagnostic performance for predicting pCR, with an area under the receiver operating characteristic curve (AUC) of 0.831 (95% confidence interval [CI]: 0.747, 0.915; *P* < 0.001). The model combining pre-treatment estrogen receptor and human epidermal growth factor receptor 2 statuses and mid-treatment ΔADC_200,1000_ improved the AUC to 0.905 (95% CI: 0.843, 0.966; *P* < 0.001).

**Conclusion:**

Mono-exponential flow-insensitive ADC change at mid-treatment was a predictor of pCR after NACT in breast cancer.

## Background

Neoadjuvant chemotherapy (NACT) has been established as one of the standard therapies for locally advanced (inoperable) or large (operable) breast cancers [1, 2]. NACT enables tumor downstaging, thus rendering inoperable tumors operable or even allowing breast-conserving surgeries. Moreover, NACT makes it possible to monitor the tumor response in vivo during treatment when compared with adjuvant chemotherapy. In particular, a pathologic complete response (pCR) after NACT has been associated with lower distant recurrence and better disease-free survival [3]. Therefore, prediction of response to NACT is crucial to optimizing treatment plan and improving individual patient-tailored management.

Noninvasive MRI plays an important role in the assessment of treatment response to NACT in breast cancer patients [4, 5]. Contrast-enhanced (CE) MRI is known as the standard imaging modality for treatment monitoring due to its high resolution and high sensitivity in breast tissues. Currently, the most widely used metric for measuring tumor change during NACT is morphologic size on CE MRI. However, changes in lesion size on breast MRI has been found to lag behind microstructural and functional alterations [6, 7].

Diffusion-weighted (DW) MRI, a functional imaging modality which reflects Brownian motion of water molecules in biologic tissues, has been extensively explored for the potential to predict therapy outcome for responders. The apparent diffusion coefficient (ADC) measured at DW MRI is commonly used to represent the magnitude of diffusion by providing information related to cellularity and the integrity of cell membranes in tumors [8–10]. Some studies have demonstrated the value of ADC in identifying responders to NACT in breast cancer patients [7, 11, 12]. However, some other studies failed to find the association between ADC and treatment response [13–15].

Many reported DW MRI studies in tissues including breast tissues have found that for a certain range of *b*-values (degree of diffusion sensitization), the diffusion signal decay presents a non-mono-exponential behavior [8, 16–18]. Therefore, conventional ADC is insufficient to reflect the complete diffusion characteristics as it is assumed on the basis of the well-behaved mono-exponential decay. Several advanced diffusion models have been proposed to reveal the complicated water molecule diffusion behavior beyond standard ADC measurements. Bi-exponential intravoxel incoherent motion (IVIM) model utilizes low *b*-values to extract the microcapillary perfusion component from the entire DW signal, while stretched-exponential model accounts for the intravoxel water diffusion heterogeneity related with microstructural complexity at high *b*-values [16]. Although bi- and stretched-exponential models have shown potential in the diagnosis and characterization of breast cancer in previous studies [19–22], their utility in predicting treatment response to NACT has not been fully understood [23, 24].

Therefore, the purpose of this study was to determine the capability of DW MRI with mono-, bi-, and stretched-exponential models in monitoring and predicting response to NACT in breast cancer patients, and further outline a model of pCR combining DW MRI parameters, CE MRI findings, and/or clinical-pathologic variables.

## Methods

### Study design and patient selection

**Fig. 1:**
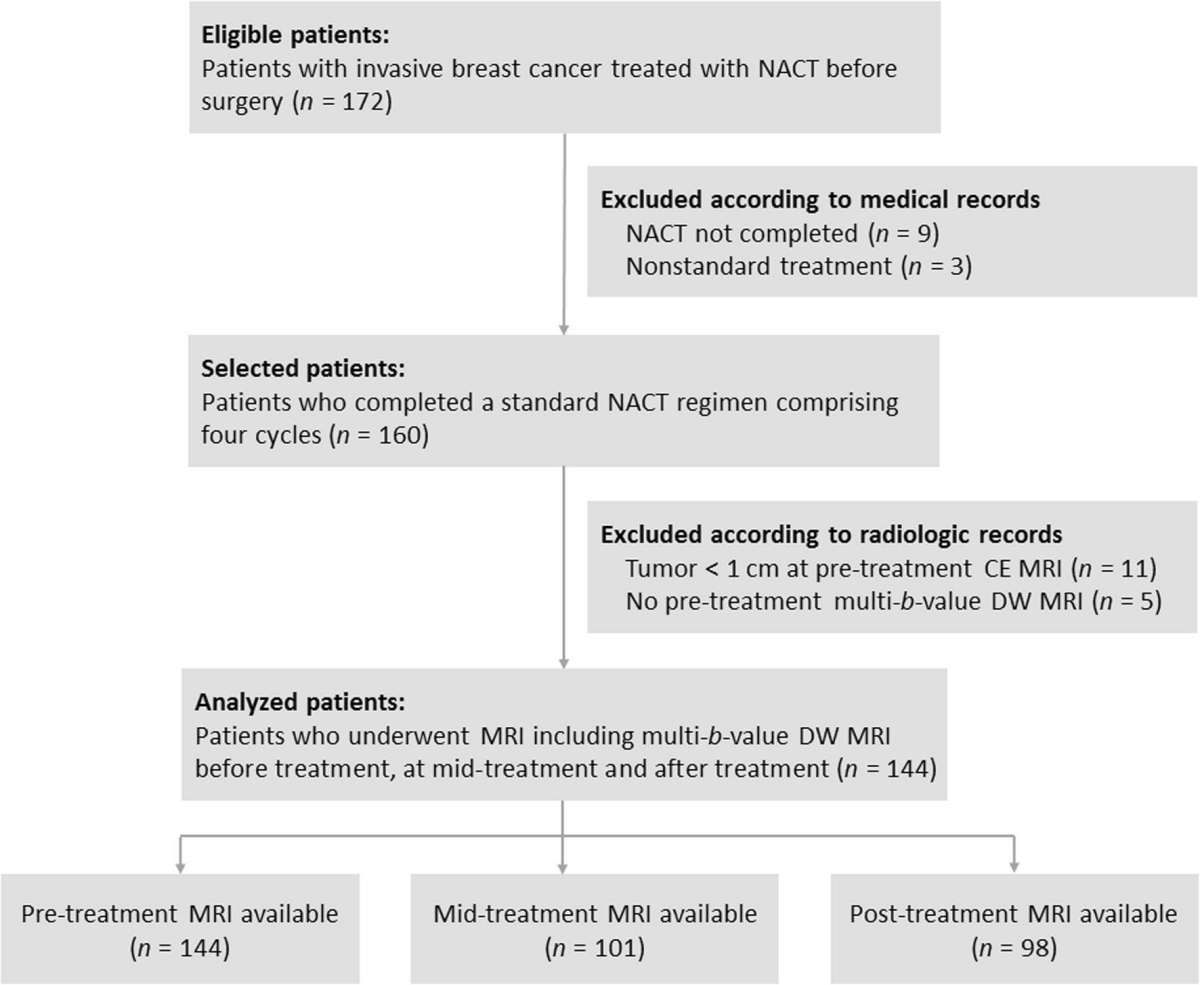
Flowchart describes inclusion and exclusion criteria

This study was approved by the Ethics Committee of Renji Hospital, School of Medicine, Shanghai Jiao Tong University, with a waiver of the requirement to obtain patient informed consent owing to the retrospective design. Subjects were identified from a retrospective review of our medical and radiologic database from November 2015 to August 2018. One hundred seventy-two women with histologically proven invasive breast cancer who received NACT as a first line of treatment were eligible for the study. The other eligibility criteria were as follows: (i) patients were aged at least 18 years old; (ii) patients were confirmed with primary breast cancer with no distant metastasis; (iii) surgical resection was preformed after completion of NACT; and (iv) MRI including multi-*b*-value DW imaging was conducted during NACT. Of the 172 patients, 28 were excluded because (i) NCAT was not completed or nonstandard treatment was used (*n* = 12); (ii) tumors were less than 1 cm at pre-treatment CE MRI (*n* = 11); and (iii) no pre-treatment multi-*b*-value DW MRI was available (*n* = 5). Therefore, 144 patients constituted the final study population (mean age, 51.7 years; age range, 25–75 years) (Fig. 1).

### Neoadjuvant chemotherapy

The treatment protocols have been previously described [25]. Each patient received intravenous administration of paclitaxel at 80 mg/m^2^ body surface area and cisplatin at 25 mg/m^2^ body surface area for four cycles lasting 16 weeks in duration. Patients with human epidermal growth factor receptor 2 (HER2)-positive findings were allowed concomitant treatment with trastuzumab, at a loading dose of 4 mg/kg body weight, followed by a maintenance dose of 2 mg/kg. Patients underwent surgery after the completion of NACT.

### MRI

Breast MRI was performed before treatment, at mid-treatment (after two cycles of NACT), and after treatment (after four cycles of NACT), prior surgery. MRI was performed by using a 3-T scanner (Ingenia; Philips Medical Systems, Best, the Netherlands) with a dedicated breast array coil. Patients were examined in the prone position. The standardized MRI protocol consisted of axial T1- and T2-weighted, sagittal fat-suppressed T2-weighted, axial fat-suppressed multi-*b*-value DW, and axial fat-suppressed dynamic CE MRI. DW images with spectral attenuated inversion recovery for fat suppression were acquired by using the single-shot echo planar imaging sequence with multiple *b*-values (0, 10, 30, 50, 100, 150, 200, 500, 800, 1000, 1500, 2000, and 2500 s/mm^2^). Other imaging parameters were: repetition time (TR), 4500 ms; echo time (TE), 85 ms; matrix, 108 × 128; in-plane resolution, 2.6 × 2.6 mm; section thickness, 3 mm; 16 sections; parallel acquisition with acceleration factor of two; and acquisition time, 8 min 40 s. Diffusion gradients were applied in three orthogonal directions. After DW imaging, dynamic CE MRI was performed by using the three-dimensional fat-suppressed T1-weighted gradient echo sequence before and after an intravenous bolus injection of 0.1 mmol/kg body weight of dimeglumine gadopentetate contrast agent (Magnevist; Bayer Healthcare, Berlin, Germany), with following parameters: TR, 4.7 ms; TE, 2.3 ms; flip angle, 10°; matrix, 320 × 340; in-plane resolution, 1.0 × 0.9 mm; section thickness, 1 mm; four or six post-contrast dynamics; and temporal resolution, 75 s.

### Image analysis

DW image analysis was performed by using custom software developed in MATLAB version R2019a (MathWorks, Natick, Mass, USA). Parametric maps for bi- and stretched-exponential models were generated by means of a nonlinear least squares fitting procedure at low (0, 10, 30, 50, 100, 150, 200, 500, and 800 s/mm^2^) and high (0, 500, 800, 1000, 1500, 2000, and 2500 s/mm^2^) *b*-values, respectively. Bi-exponential pseudodiffusion coefficient D* and perfusion fraction *f*, and stretched-exponential distributed diffusion coefficient DDC and intravoxel heterogeneity index *α* were calculated. For mono-exponential modeling, all *b*-value were used to fit the ADC_all_ maps. Standard ADC maps were also calculated using two *b*-values. Specifically, *b* values of 0 and 1000 s/mm^2^ were included to obtain the routinely used standard ADC (ADC_0,1000_), and 200 and 1000 s/mm^2^ to obtain the flow-insensitive ADC (ADC_200,1000_) [21, 26].

Region of interest (ROI) delineation was performed by a radiologist with 10 years of experience in interpretation of breast MR images. ROIs encompassing the entire tumor were manually drawn on all sections of high *b*-value DW images. Tumor areas were defined as hyperintensity on DW images by avoiding T2 shine-through regions (eg, cystic and necrotic components). CE MRI was used for lesion localization and boundary verification. ROIs were then transferred to corresponding parametric maps, and mean values of all voxels within the ROIs were calculated. Tumor ROIs at each treatment time point were identified by referencing the lesion location on prior MRI examinations. If no residual enhanced tumor areas appeared on post-treatment CE MRI, ROIs were placed in the same region as the last positive MRI [27].

On CE MRI, the longest diameter (size) and relative enhancement ratio (RER) of the tumor was measured. RER was defined as $\left[ {\left( {SI{_{post}} - \, SI_{pre} } \right)/SI{_{pre}} } \right] \times 100$ , where *SI*_*pre*_ is the CE MRI signal intensity of the tumor before contrast injection and *SI*_*post*_ is the signal intensity of the first post-contrast dynamic acquisition [28].

### Molecular biomarkers

Statuses for estrogen receptor (ER), progesterone receptor (PR), HER2 and Ki-67 labeling index were determined from pre-treatment biopsy by immunohistochemistry (IHC). ER or PR positivity was defined as ≥ 1% nuclear immunostaining. HER2 expression was deemed as positive when membrane immunostaining was scored 3+ or 2+ with an amplification of HER2 gene demonstrated by in situ hybridization assays. Ki-67 index was assessed as the percentage of immunoreactive tumor cells, and a cut-off value of 20% was used to define the low- and high-proliferation tumor groups [29].

### Pathologic response analysis

The final histopathologic examination was performed after surgical resection following the last cycle of NACT, and the findings were considered as the reference standard for determining the reliability of DW MRI for predicting the treatment response in our study. Patients were categorized as having a pCR if no residual invasive tumor existed in the surgical specimen with the absence of axillary lymph node invasion, regardless of the presence of ductal carcinoma in situ (DCIS).

### Statistical analysis

Continuous variables were expressed as means ± standard deviations, and categorical variables as numbers and percentages. Clinical-pathologic characteristics were compared according to response to NACT by using the *t* test, *χ*^*2*^ test, or Fisher exact test, where appropriate. Quantitative MRI findings were initially screened for normality using the Shapiro Wilk test. Comparisons between pCR and non-pCR groups were made with independent samples *t* test for normally distributed variables or Wilcoxon rank sum test for non-normally distributed variables. Receiver operating characteristic (ROC) curves were generated to test the predictive ability for pCR by using the area under the ROC curve (AUC) and its 95% confidence interval (CI). Youden index was used to identify the optimal threshold.

Univariate and multivariate logistic regression analyses were performed to screen the independent clinical-pathologic and imaging predictors of pCR. Variables with a *P* value < 0.05 at univariate analysis were fed into multivariate backward stepwise logistic regression analysis. Logistic regression coefficients were exponentiated to obtain odds ratios and 95% Cls. ROC curve was constructed to calculate AUC along with its 95% Cl for the predictive model. The method of DeLong et al. [30] was used for statistical comparison of AUCs between the multivariate model and univariate predictors. Leave-one-out cross-validation was applied to evaluate the performance of the predictive model, and the corresponding sensitivity, specificity and accuracy were determined. *P* value < 0.05 was considered statistically significant, except for those in which a Bonferroni correction was performed for multiple comparison. Bonferroni-adjusted significance level was set at *P* value < 0.0024 (0.05/21) for DW imaging variables (seven variables and three time points) and at *P* value < 0.0083 (0.05/6) for CE MRI variables (two variables and three time points). Statistical analyses were carried out using SPSS version 21 (IBM SPSS Statistics, Armonk, New York, USA) and GraphPad Prism 5 (GraphPad Software, La Jolla, California, USA).

## Results

### Patient characteristics

Patient characteristics are listed in Table 1. Of the 144 patients, 54 (37.5%) achieved pCR at final histopathologic examination. In terms of molecular biomarkers, the pCR group showed a significant higher proportion of ER negativity (35 of 54 patients [64.8%]; *P* < 0.001), PR negativity (24 of 54 patients [44.4%]; *P* = 0.005), HER2 positivity (33 of 54 patients [61.1%]; *P* = 0.001), and Ki-67 ≥ 20% (52 of 54 patients [96.3%]; *P* < 0.001). No significant difference was observed in age (*P* = 0.876), menopausal status (*P* = 0.334), or tumor histologic type (*P* = 0.535) between the pCR and non-pCR groups.

**Table 1:** Patient characteristics

Characteristic	All patients	Patients with pCR	Patients with non-pCR	*P* value
No. of patients	144	54 (37.5)	90 (62.5)	
Age (y)				
Mean ± standard deviation	51.7 ± 11.8	51.5 ± 12.0	51.9 ± 11.7	0.876
Range	25–75	26–73	25–75	
Menopause				
Premenopausal	58 (40.3)	19 (35.2)	39 (43.3)	0.334
Postmenopausal	86 (59.7)	35 (64.8)	51 (56.7)	
Histologic type				
IDC	132 (91.7)	51 (94.4)	81 (90.0)	0.535
Non-IDC	12 (8.3)	3 (5.6)	9 (10.0)	
Estrogen receptor				
Negative	53 (36.8)	35 (64.8)	18 (20.0)	< 0.001
Positive	91 (63.2)	19 (35.2)	72 (80.0)	
Progesterone receptor				
Negative	44 (30.6)	24 (44.4)	20 (22.2)	0.005
Positive	100 (69.4)	30 (55.6)	70 (77.8)	
Human epidermal growth factor receptor 2				
Negative	81 (56.3)	21 (38.9)	60 (66.7)	0.001
Positive	63 (43.8)	33 (61.1)	30 (33.3)	
Ki-67				
< 20%	26 (18.1)	2 (3.7)	24 (26.7)	< 0.001
≥ 20%	118 (81.9)	52 (96.3)	66 (73.3)	

Unless otherwise noted, values are numbers of patients, with percentages in parentheses. Percentages may not add up to 100% because of rounding

IDC: invasive ductal carcinoma; pCR: pathologic complete response

### DW MRI findings

**Fig. 2:**
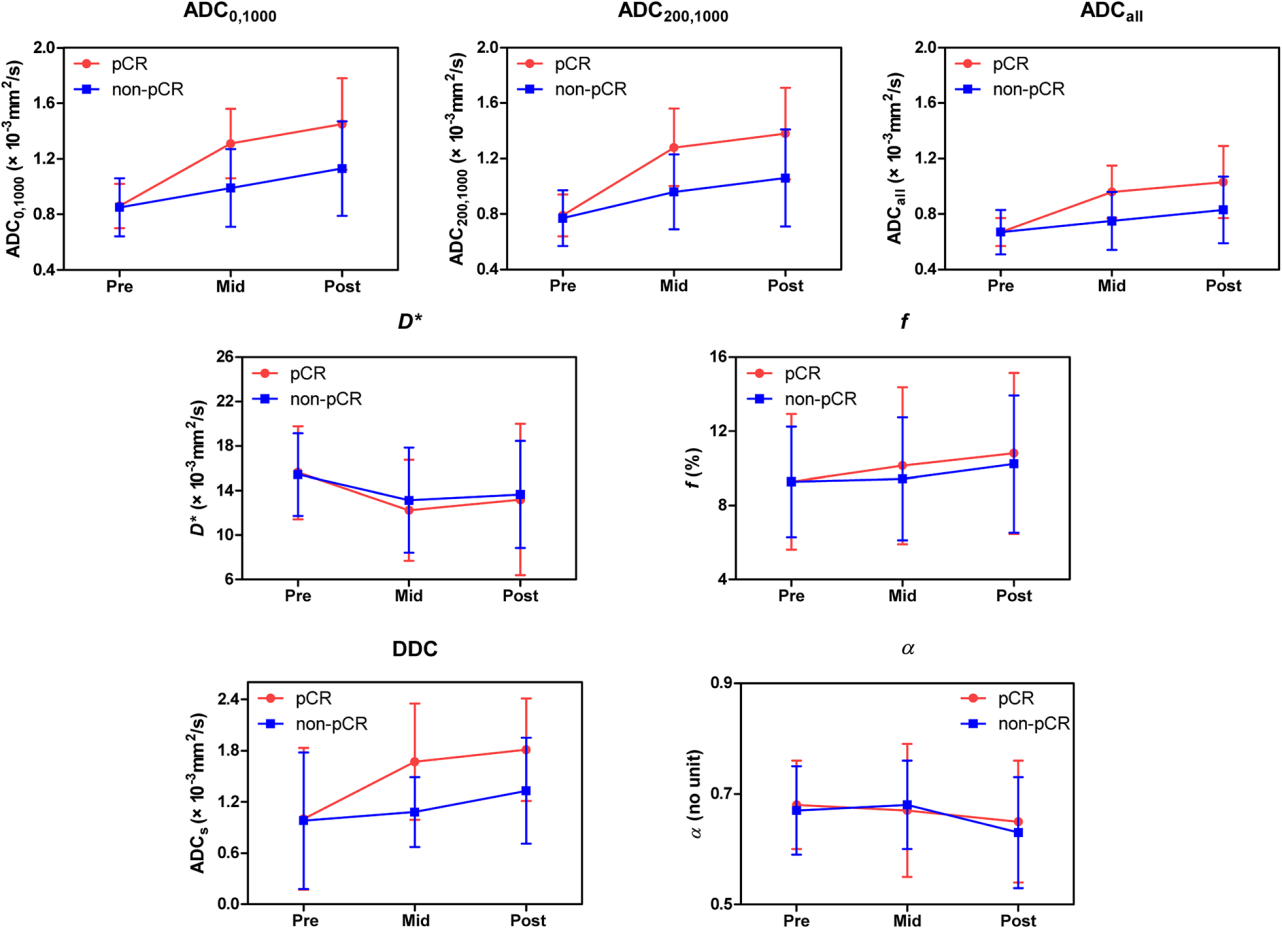
Time courses of mono-, bi-, and stretched-exponential diffusion-weighted imaging measures during neoadjuvant chemotherapy. Shown are mean values from all available subjects, with error bars indicating standard deviations. A discrepancy in parameter variations was recorded between groups with and without pathologic complete response, especially for diffusion-related imaging measures including ADC_0,1000_, ADC_200,1000_, ADC_all_, and DDC

**Fig. 3:**
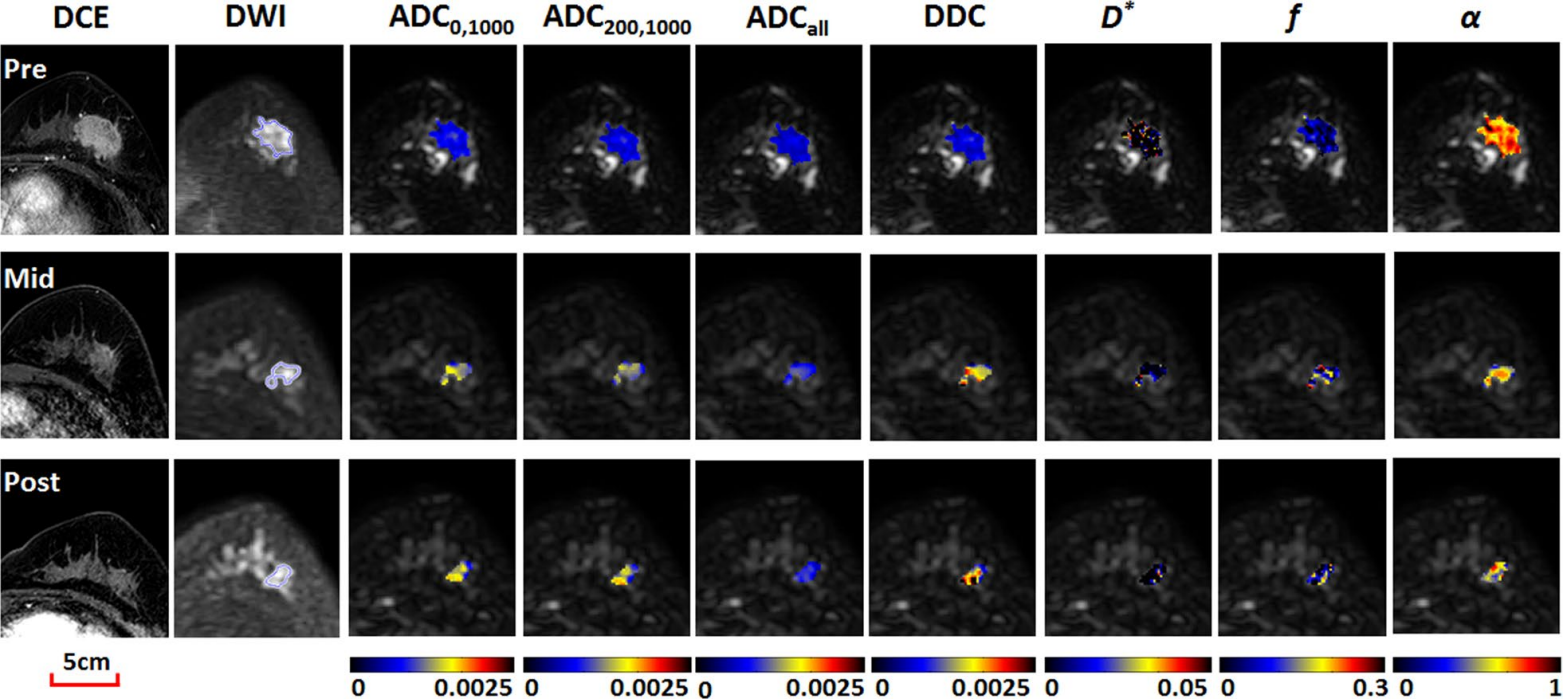
Images obtained in a 56-year-old woman with invasive ductal carcinoma (estrogen receptor-/progesterone receptor-/human epidermal growth factor receptor 2 + /Ki-67 > 20%) who was categorized in the pathologic complete response group. Shown from left to right are contrast-enhanced MRI images, diffusion-weighted (DW) MRI images (*b* = 1500 s/mm^2^), and color-coded quantitative DW MRI maps (overlaid on original DW images). Serial diffusion-related measures increased progressively with treatment. For example, ADC_200,1000_ values were 0.68, 0.97 and 1.08 × 10^–3^ mm^2^/s, respectively, at pre-, mid-, and post-treatment

**Fig. 4:**
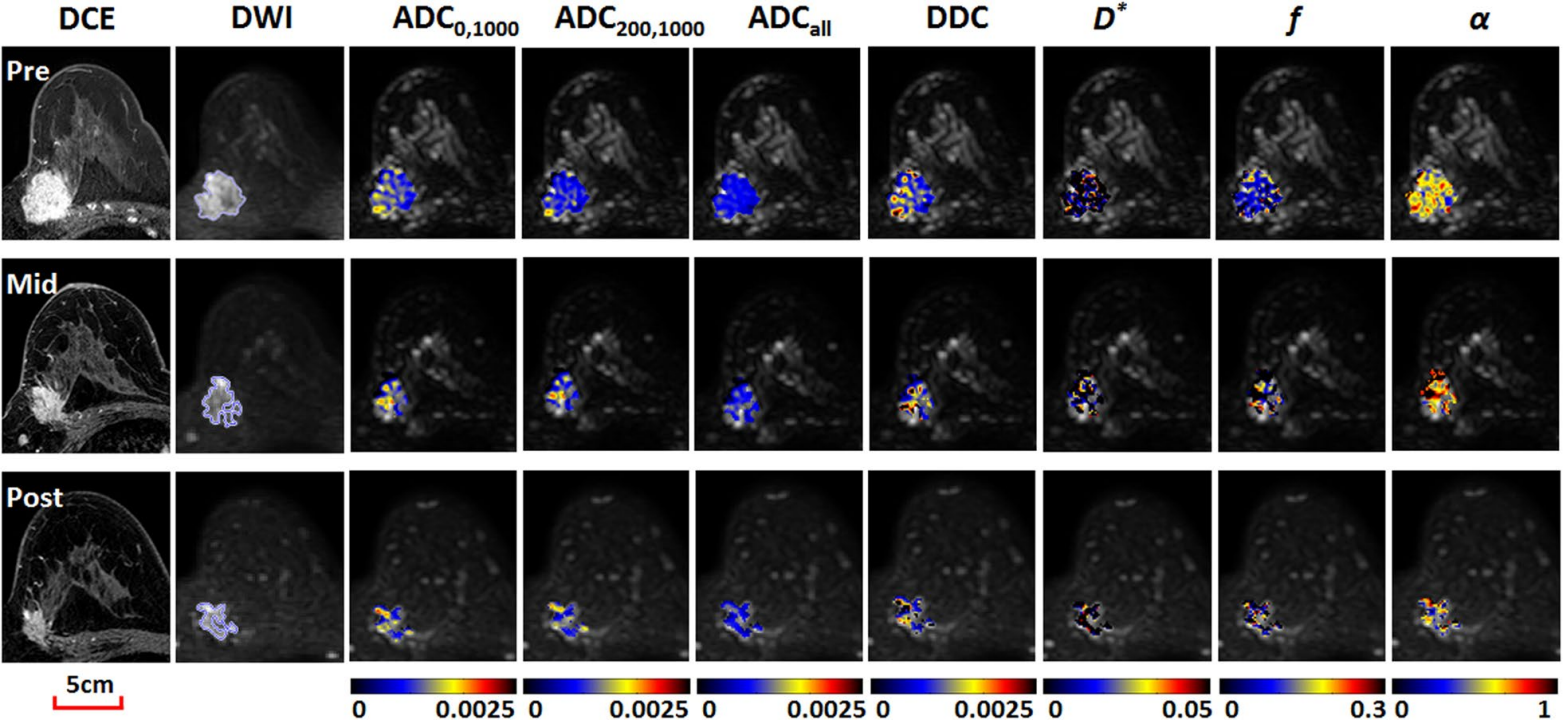
Images obtained in a 49-year-old woman with invasive ductal carcinoma (estrogen receptor+/progesterone receptor+/human epidermal growth factor receptor 2-/Ki-67 > 20%) who was categorized in the non-pathologic complete response group. Shown from left to right are contrast-enhanced MRI images, diffusion-weighted (DW) MRI images (*b* = 1500 s/mm^2^), and color-coded quantitative DW MRI maps (overlaid on original DW images). Serial diffusion-related measures showed no obvious increase with treatment. For example, ADC_200,1000_ values were 0.86, 0.89 and 0.80 × 10^–3^ mm^2^/s, respectively, at pre-, mid-, and post-treatment

All pre-treatment DW imaging measures showed no significant differences between patients with and those without pCR (Table 2). The time courses of diffusion-related imaging measures including ADC_0,1000_, ADC_200,1000_, ADC_all_, and DDC represented a generally increasing trend as treatment progressed, and the extent of changes during treatment differed between the pCR and non-pCR groups (Fig. 2). Examples of dynamic changes of DW imaging measures in the pCR and non-pCR groups during NACT are shown in Figs. 3 and 4. Statistical results showed that ΔADC_0,1000_, ΔADC_200,1000_, ΔADC_all_, and ΔDDC were greater in patients with pCR than in patients without pCR at mid-treatment or post-treatment (*P* ≤ 0.001). However, there were no significant differences in ΔD*, Δ*f*, or Δ*α* between the two groups at any time point (adjusted *P* > 0.0024). Among the significant measures, ΔADC_200,1000_ at mid-treatment exhibited the highest diagnostic performance for predicting pCR, with an AUC of 0.831 (95% CI: 0.747, 0.915; *P* < 0.001) (Table 2).

**Table 2:** Diffusion-weighted MRI findings according to response at each time point

Variable (× 10^–3^ mm^2^/s)	Patients with pCR	Patients with non-pCR	*P* value^*^	AUC	95% CI	*P* value^*^
Pre-treatment						
No. of patients	54	90				
ADC_0,1000_	0.86 ± 0.16	0.85 ± 0.21	0.324	0.549	0.455, 0.644	0.324
ADC_200,1000_	0.79 ± 0.15	0.77 ± 0.20	0.115	0.579	0.484, 0.673	0.115
ADC_all_	0.67 ± 0.10	0.67 ± 0.16	0.302	0.551	0.458, 0.645	0.302
D*	15.62 ± 4.18	15.44 ± 3.70	0.794	0.502	0.402, 0.601	0.974
* f* ^a^	9.27 ± 3.66	9.27 ± 2.98	0.729	0.517	0.417, 0.618	0.729
DDC	1.00 ± 0.83	0.98 ± 0.80	0.478	0.535	0.440, 0.630	0.478
* α*^a^	0.68 ± 0.08	0.67 ± 0.08	0.306	0.551	0.452, 0.650	0.306
Mid-treatment						
No. of patients	37	64				
ΔADC_0,1000_	0.46 ± 0.26	0.15 ± 0.23	< 0.001	0.812	0.727, 0.897	< 0.001
ΔADC_200,1000_	0.50 ± 0.26	0.19 ± 0.22	< 0.001	0.831	0.747, 0.915	< 0.001
ΔADC_all_	0.28 ± 0.20	0.09 ± 0.17	< 0.001	0.749	0.640, 0.858	< 0.001
ΔD^*^	− 3.06 ± 6.36	− 1.97 ± 6.35	0.409	0.577	0.460, 0.694	0.197
Δ*f* ^a^	1.78 ± 4.33	0.82 ± 3.86	0.406	0.550	0.432, 0.667	0.406
ΔDDC	0.78 ± 0.68	0.25 ± 0.35	< 0.001	0.778	0.675, 0.882	< 0.001
Δ*α*^a^	− 0.02 ± 0.12	− 0.003 ± 0.09	0.478	0.503	0.364, 0.641	0.964
Post-treatment						
No. of patients	33	65				
ΔADC_0,1000_	0.60 ± 0.34	0.30 ± 0.32	< 0.001	0.743	0.637, 0.849	< 0.001
ΔADC_200,1000_	0.60 ± 0.32	0.31 ± 0.32	< 0.001	0.745	0.641, 0.849	< 0.001
ΔADC_all_	0.35 ± 0.26	0.18 ± 0.22	0.001	0.693	0.574, 0.813	0.003
ΔD^*^	− 1.84 ± 7.71	− 1.47 ± 5.90	0.794	0.528	0.402, 0.653	0.655
Δ*f* ^a^	2.90 ± 5.33	1.69 ± 3.73	0.482	0.544	0.410, 0.677	0.482
ΔDDC	0.96 ± 0.63	0.51 ± 0.60	0.001	0.726	0.610, 0.841	< 0.001
Δ*α*^a^	− 0.05 ± 0.12	− 0.05 ± 0.10	0.820	0.503	0.368, 0.637	0.964

^a^*f* and *α* have no units

^*^P < 0.0024 (0.05/21) is defined as the Bonferroni-corrected significance level

ADC: apparent diffusion coefficient, D^*^: pseudodiffusion coefficient, *f*: perfusion fraction, DDC: distributed diffusion coefficient, *α*: intravoxel heterogeneity index, pCR: pathologic complete response, AUC: area under the receiver operating characteristic curve, CI: confidence interval

### CE MRI findings

Similarly, pre-treatment tumor size or RER on CE MRI did not differ significantly between patients with and those without pCR (adjusted *P* > 0.0083). By mid-treatment, tumor size and RER showed greater changes in patients with pCR than in patients without pCR (*P* ≤ 0.001), with predictive AUC of 0.698 (95% CI: 0.591, 0.804; *P* = 0.001) and 0.706 (95% CI: 0.603, 0.809; *P* = 0.001), respectively. ΔRER at post-treatment also predictive of pCR (AUC = 0.734; 95% CI: 0.633, 0.836; *P* < 0.001) (Table 3).

**Table 3:** Contrast-enhanced MRI findings according to response at each time point

Variable	Patients with pCR	Patients with non-pCR	*P* value	AUC	95% CI	*P* value
Pre-treatment						
No. of patients	54	90				
Size (mm)	39.8 ± 21.2	44.3 ± 18.8	0.189	0.578	0.475, 0.681	0.119
RER (%)	164.1 ± 66.5	152.6 ± 57.0	0.271	0.551	0.452, 0.650	0.305
Mid-treatment						
No. of patients	37	64				
ΔSize (mm)	− 27.4 ± 16.6	− 17.1 ± 14.2	0.001	0.698	0.591, 0.804	0.001
ΔRER (%)	− 101.2 ± 81.1	− 36.8 ± 81.1	< 0.001	0.706	0.603, 0.809	0.001
Post-treatment						
No. of patients	33	65				
ΔSize (mm)	− 34.0 ± 18.9	− 24.6 ± 18.5	0.021	0.661	0.549, 0.774	0.009
ΔRER (%)	− 135.1 ± 81.4	− 60.5 ± 79.8	< 0.001	0.734	0.633, 0.836	< 0.001

^*^P < 0.0083 (0.05/6) is defined as the Bonferroni-corrected significance level

RER: relative enhancement ratio; pCR: pathologic complete response; AUC: area under the receiver operating characteristic curve; CI confidence interval

### Logistic regression modeling

**Fig. 5:**
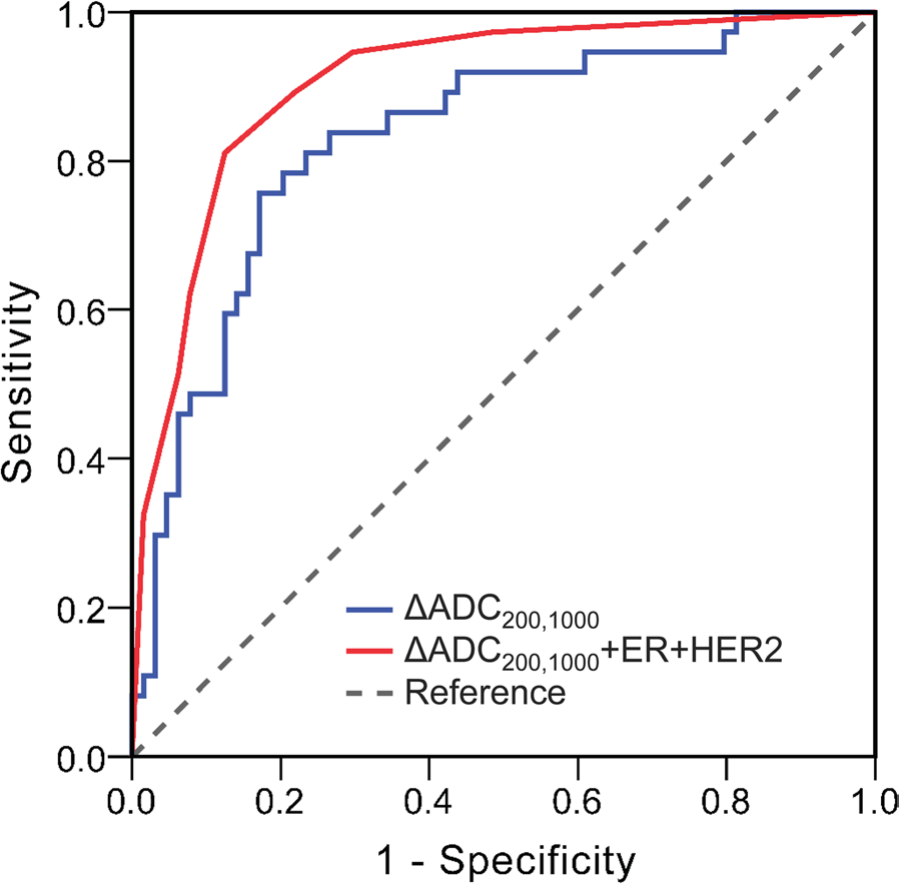
Receiver operating characteristic curves for mid-treatment ADC_200,1000_ change (ΔADC_200,1000_) and regression model (ΔADC_200,1000_ + estrogen receptor [ER] + human epidermal growth factor receptor 2 [HER2]) as predictors of pathologic complete response. The areas under the receiver operating characteristic curves were 0.831 (95% confidence interval: 0.747, 0.915) and 0.905 (95% confidence interval: 0.843, 0.966), respectively

Pre-treatment clinical-pathologic and mid-treatment imaging variables were used to construct a predictive model by logistic regression analysis (Table 4). Mid-treatment ΔADC_200,1000_, ΔSize, and ΔRER were stratified into binary categorical variables using the optimal threshold of 0.33 × 10^–3^ mm^2^/s, -26.6 mm, and -87.5%, respectively, according to the ROC curve analysis. In the univariate regression analysis, ER negativity (*P* < 0.001), HER2 positivity (*P* < 0.001), Ki-67 ≥ 20% (*P* = 0.012), ΔADC_200,1000_ > 0.33 × 10^–3^ mm^2^/s (*P* < 0.001), ΔSize ≤ − 26.6 mm (*P* < 0.001), and ΔRER ≤ -87.5% (*P* < 0.001) were found to be associated with a higher probability of achieving pCR. In the multivariate analysis, however, only ER negativity (odds ratio, 11.433; 95% CI: 3.363, 38.874; *P* < 0.001), HER2 positivity (odds ratio, 5.469; 95% CI: 1.631, 18.339; *P* = 0.006), and ΔADC_200,1000_ > 0.33 × 10^–3^ mm^2^/s (odds ratio, 9.074; 95% CI: 2.847, 28.917; *P* < 0.001) remained significant independent factors for pCR (Table 4). The regression model combining these three variables resulted in an overall predictive performance of AUC = 0.905 (95% CI: 0.843, 0.966; *P* < 0.001), which was greater than the AUC of ΔADC_200,1000_ alone, with a near-significant difference (*P* = 0.060) (Fig. 5). By using leave-one-out cross validation, the multivariate model achieved a sensitivity of 81.1%, a specificity of 87.5%, and an accuracy of 85.1% for predicting pCR.

**Table 4:** Univariate and multivariate analyses of variables associated with pCR

Variable	Univariate analysis		Multivariate analysis	
	Odds ratio^*^	*P* value	Odds ratio^*^	*P* value
Pre-treatment				
Age	0.986 (0.953, 1.021)	0.429	–	–
Menopause (post- vs. pre-menopausal)	2.083 (0.895, 4.850)	0.089	–	–
Histologic type (IDC vs. Non-IDC)	1.770 (0.177, 17.665)	0.626	–	–
ER (negative vs. positive)	11.388 (4.367, 29.698)	< 0.001	11.433 (3.363, 38.874)	< 0.001
PR (negative vs. positive)	2.388 (0.970, 5.881)	0.058	–	–
HER2 (positive vs. negative)	5.104 (2.131, 12.227)	< 0.001	5.469 (1.631, 18.339)	0.006
Ki-67 (≥ 20% vs. < 20%)	14.087 (1.795, 110.566)	0.012	3.852 (0.299, 49.574)	0.301
Mid-treatment				
ΔADC_200,1000_ (> 0.33 vs. ≤ 0.33 × 10^–3^ mm^2^/s)	13.481 (5.066, 35.877)	< 0.001	9.074 (2.847, 28.917)	< 0.001
ΔSize (≤ − 26.6 vs. > − 26.6 mm)	5.098 (2.070, 12.553)	< 0.001	2.351 (0.697, 7.931)	0.168
ΔRER (≤ − 87.5% vs. > -87.5%)	4.202 (1.748, 10.100)	0.001	1.231 (0.339, 4.467)	0.752

^*^Data in parentheses are 95% confidence intervals

For binary categorical variables, the latter category in the parentheses was used as the reference

IDC: invasive ductal carcinoma; pCR: pathologic complete response; ER: estrogen receptor; PR: progesterone receptor; HER2: human epidermal growth factor receptor 2; ADC: apparent diffusion coefficient; RER: relative enhancement ratio

## Discussion

Identification of breast cancer patients who will benefit from NACT and achieve a final pCR is pivotal. Our study showed that mid-treatment flow-insensitive ADC changes were capable of predicting tumor treatment response. Patients with pre-treatment ER negativity and HER2 positivity, and greater mid-treatment ADC changes had more potential to achieve pCR after NACT. Advanced diffusion models including bi- and stretched-exponential models showed no additional benefit for the prediction.

Our results are concordant with those from other studies [27, 31], indicating that mid-treatment ADC changes might be predictive of pCR. Greater mid-treatment increases in tumor ADC from baseline were demonstrated in responders versus nonresponders. The increase in ADC values after NACT is believed to be a consequence of apoptosis and cell necrosis induced by chemotherapy [27]. Responders are more chemosensitive, thus resulting in more reduction in tumor cellularity and cell membrane integrity, reflected by greater ADC increases during treatment. Among all the ADC metrics analyzed in the study, the mono-exponential model derived ADC with *b*-values of 200 and 1000 s/mm^2^ exhibited a superior prediction performance. According to the IVIM theory, the contribution of microcirculation-related pseudodiffusion on DW MRI signal is almost negligible at high *b*-values (e.g., > 200 s/mm^2^). Therefore, flow-insensitive ADC_200,1000_ is merely accounted for by pure water molecule diffusion, which is thought to have a more direct association with tissue cellularity and cell membrane integrity. From another aspect, it can be implied that diffusion may outperform perfusion in predicting the treatment response of NACT in breast cancer. This implication can also be confirmed by our results from bi-exponential IVIM and CE MRI analysis, showing that IVIM based pseudodiffusion coefficient D^*^ and perfusion fraction *f*, and CE MRI based relative enhancement ratio RER were all inferior to ADC metrics for the prediction.

Like previous studies [12, 13, 27, 32], pre-treatment ADC values were not predictive of NACT response in our cohort. This accordance can partly be attributed to the same type of reference standard used in these studies and ours, that is, responders and nonresponders were categorized by means of final histopathologic assessment. In the studies of Santamaria et al. [12] and Woodhams et al. [13], pCR was defined as the complete absence of any residual invasive cancer or DCIS, while in the studies of Partridge et al. [27], Fangberget et al. [32], and ours, pCR was defined as the complete absence of invasive cancer of any size, regardless of DCIS. Though the definition of pCR is slightly different among these studies, all reported ADC values prior to therapy did not predict pCR. Some other studies used clinical response (tumor size shrinkage on radiologic examination) as the reference standard and conflicting results have been demonstrated [33, 34]. For example, Park et al. [33] and Sharma et al. [34] showed that pre-treatment ADC values had predictive value of clinical therapeutic response, with clinical responders representing substantially lower pre-treatment ADC values compared with nonresponders.

Predictive value of bi- and stretched-exponential DW MRI in assessing treatment response of NACT in breast cancer is rarely investigated. The results of this study demonstrated no significant benefit of bi-exponential (D^*^, *ƒ*) and stretched-exponential (DDC, *α*) parameters for predicting pCR as compared with mono-exponential ADC. Changes of bi-exponential (D^*^, *ƒ*) and stretched-exponential (*α*) parameters during NACT were not significantly different between responders and nonresponders, concordant with the results of prior studies of Bedair et al. [23] and Kim et al. [35]. The limited value of bi-exponential D^*^ and *ƒ* may be explained by their high estimation uncertainty due to the non-linearity of the bi-exponential model [35, 36]. Though stretched-exponential *α* is proposed to be a heterogeneity index of water diffusion environment, its underlying biologic basis still remains unclear. Likewise, in a recent study on response assessment of liver metastases to chemotherapy in colorectal cancer, the usefulness of *α* value was also not identified [37].

In clinical settings, treatment response is mostly evaluated using tumor size alteration according to the RECIST criteria. However, our results showed that tumor size measured on CE MRI was less useful than ADC for predicting treatment response to NACT. This finding is consistent with that of a previous study showing that ADC change after the first cycle of NACT in breast cancer was statistically significant compared with volume and diameter, even though clinical response criteria were used as the reference standard in the study 7. Breast CE MRI provides an additional tool for assessment of tumor size. However, therapy-induced changes may cause substantial over- or under-estimation of tumor size, especially in well-responding tumors [38]. Therefore, tumor shrinkage on CE MRI may be not an exact reflection of the true histologic regression status. In addition, it is also believed that morphologic changes often occur relatively late and thus may not accurately assess early tumor response during the time course of NACT [7, 31].

In this study, breast cancer with ER/PR negativity, HER2 positivity or Ki-67 ≥ 20% was more likely to reach a pCR after NACT. This finding has already been recognized [39, 40] and probably a higher cellular proliferation of these tumor types renders tumor cells more sensitive to chemotherapy. Multivariate logistic regression analysis suggested that ER negativity, HER2 positivity and mid-treatment ΔADC_200,1000_ > 0.33 × 10^–3^ mm^2^/s were the significant predictors. ROC analysis indicated a better predicting performance when all the three variables were included in the model, with an AUC of 0.905. This is in agreement with the results published by Santamaría et al. [12], who found the model incorporating breast cancer subtype and MRI features (including ADC ratio after treatment) demonstrated a higher accuracy relative to prediction of pCR with an AUC of 0.92.

Our study had limitations. First, this was a retrospective study in a single institution. Patient selection bias may exist. Second, due to the retrospective design, MRI was not performed during early treatment, so an evaluation of the early response to NACT was not possible. The role of ΔADC in prediction of pCR at early treatment, however, remains controversial in the literature [7, 27]. Third, the interobserver variability or reproducibility of quantitative DW MRI measurements was not evaluated. However, we calculated the tumor DW MRI parameters over the entire tumor volume delineated by one experienced in breast MRI. In addition, the interobserver agreement of mono-, bi-, and stretched-exponential DW MRI parameters has been demonstrated to be good to excellent in our previous studies [20, 41]. Fourth, quantitative DW MRI parameters were measured by averaging all voxels within the ROI. More comprehensive analytic methods may provide added-value information. For example, histogram and texture analyses highlight the different heterogeneous appearances of breast cancer on ADC maps, which have proved to be related with tumor biology [42, 43].

## Conclusions

In conclusion, mono-exponential flow-insensitive ADC change at mid-treatment was the most accurate predictor of pCR to neoadjuvant chemotherapy in patients with breast cancer among all parameters from mono-, bi-, and stretched-exponential DW MRI models. Patients with pre-treatment ER negativity and HER2 positivity, and greater mid-treatment ADC changes are more likely to completely respond to neoadjuvant chemotherapy. If these results are validated in future studies with larger cohort in more institutions, it could help guide the treatment and predict the long-term outcome.

## Data Availability

The datasets used and/or analysed during the current study are available from the corresponding author on reasonable request.

## Abbreviations

DW: Diffusion-weighted
pCR: Pathologic complete response
NACT: Neoadjuvant chemotherapy
ADC: Apparent diffusion coefficient
CE: Contrast-enhanced
IVIM: Intravoxel incoherent motion
HER2: Human epidermal growth factor receptor 2
TR: Repetition time
TE: Echo time
*D*^***^: Pseudodiffusion coefficient
*f*: Perfusion fraction
DDC: Distributed diffusion coefficient
*α*: Intravoxel heterogeneity index
ROI: Region of interest
RER: Relative enhancement ratio
ER: Estrogen receptor
PR: Progesterone receptor
IHC: Immunohistochemistry
DCIS: Ductal carcinoma in situ
ROC: Receiver operating characteristic
AUC: Area under the receiver operating characteristic curve
CI: Confidence interval
